# Exhaled carbon monoxide and its associations with smoking, indoor household air pollution and chronic respiratory diseases among 512 000 Chinese adults

**DOI:** 10.1093/ije/dyt158

**Published:** 2013-09-20

**Authors:** Qiuli Zhang, Liming Li, Margaret Smith, Yu Guo, Gary Whitlock, Zheng Bian, Om Kurmi, Rory Collins, Junshi Chen, Silu Lv, Zhigang Pang, Chunxing Chen, Naying Chen, Youping Xiong, Richard Peto, and Zhengming Chen

**Affiliations:** ^1^Clinical Trial Service Unit and Epidemiological Studies Unit (CTSU), University of Oxford, Oxford, UK, ^2^School of Public Health, Peking University Health Science Center, Beijing, China, ^3^Chinese Academy of Medical Sciences, Dong Cheng District, Beijing, China, ^4^China National Center For Food Safety Risk Assessment, Chaoyang District, Beijing, China, ^5^Licang Center for Disease Control and Prevention, Qingdao, Shandong, China, ^6^Heilongjiang Center for Disease Control and Prevention, Harbin, Heilongjiang, China, ^7^Meilan Center for Disease Control and Prevention, Haikou, Hainan, China, ^8^Guangxi Center for Disease Control and Prevention, Nanning, Guangxi, China and ^9^Liuyang Center for Disease Control and Prevention, Changsha, Hunan, China

**Keywords:** Exhaled carbon monoxide, smoking, household air pollution, epidemiology, China

## Abstract

**Background** Exhaled carbon monoxide (COex) level is positively associated with tobacco smoking and exposure to smoke from biomass/coal burning. Relatively little is known about its determinants in China despite the population having a high prevalence of smoking and use of biomass/coal.

**Methods** The China Kadoorie Biobank includes 512 000 participants aged 30-79 years recruited from 10 diverse regions. We used linear regression and logistic regression methods to assess the associations of COex level with smoking, exposures to indoor household air pollution and prevalent chronic respiratory conditions among never smokers, both overall and by seasons, regions and smoking status.

**Results** The overall COex level (ppm) was much higher in current smokers than in never smokers (men: 11.5 vs 3.7; women: 9.3 vs 3.2). Among current smokers, it was higher among those who smoked more and inhaled more deeply. Among never smokers, mean COex was positively associated with levels of exposures to passive smoking and to biomass/coal burning, especially in rural areas and during winter. The odds ratios (OR) and 95% confidence interval (CI) of air flow obstruction (FEV_1_/FVC ratio <0.7) for never smokers with COex at 7–14 and ≥14 ppm, compared with those having COex <7, were 1.38 (1.31–1.45) and 1.65 (1.52–1.80), respectively (*P*_trend_ <0.001). Prevalence of other self-reported chronic respiratory conditions was also higher among people with elevated COex (*P* <0.05).

**Conclusion** In adult Chinese, COex can be used as a biomarker for assessing current smoking and overall exposure to indoor household air pollution in combination with questionnaires.

## Introduction

The carbon monoxide level in exhaled breath (COex) can be measured easily and cheaply using portable meters that are now used both in clinical practice and in research studies.^1^^,^^2^ Evidence from Europe,^3^^,^^4^ North America^5^^,^^6^ and elsewhere^7–9^ shows that COex levels are on average much higher in smokers than non-smokers, so such measurements have been used as biomarkers for assessing smoking exposure in smoking cessation programmes^10^^,^^11^ and in clinical, laboratory and epidemiological studies.^7^^,^^12^ Despite the country’s very high prevalence of smoking among males relatively little is known about the lifestyle and environmental determinants of COex levels in China; over half of Chinese men still smoke,^13^ and about one-third of the world’s male smokers now live in China.^14^ There are also distinctive features about smoking in China which mean that findings cannot simply be extrapolated from other countries and areas: for example, there are relatively high prevalences of occasional smoking and passive smoking in China.^13^ Use of biomarkers such as COex to help validate self-reported smoking status in epidemiological studies will be important for reliable assessment of present and future hazards associated with smoking in China and other developing countries. Moreover, many people still live in areas with really high levels of exposure to indoor air pollution which could potentially also affect COex level.^15^^,^^16^

To help fill these gaps in knowledge with respect to China, we analysed cross-sectional data on COex level, smoking habits and household air pollution in a study of over 510 000 adults in 10 diverse regions of China, from the China Kadoorie Biobank study (CKB).^17^ The main aims of this paper are to determine the relevance to COex level of smoking habits, household air pollution and location of residence, and also to assess the associations of COex level with the prevalence of certain respiratory diseases and related symptoms in never smokers.

## Materials and Methods

### Baseline survey

Details of the CKB study design, methods and participants have been described previously.^17^^,^^18^ Briefly, over 512 000 adults aged 30–79 years were enrolled into CKB during 2004–08 from 10 geographically defined areas across China (five urban and five rural). The areas were chosen according to local disease patterns, exposure to certain risk factors, population stability, quality and coverage of death and disease registries, and local capacity and long-term commitment. At the baseline survey, a laptop-based questionnaire was administered by an interviewer to each participant. The questionnaire consisted of 10 major sections: general demographic and socio-economic status, diet and lifestyle habits (e.g. smoking, alcohol drinking and tea drinking), work and leisure-related physical activity, exposure to passive smoking and household air pollution (e.g. frequency of cooking, types of fuel and ventilation used for cooking and heating), medical history and current medication use and (in women) reproductive history. Smoking information was collected both about its usual patterns on a typical day (e.g. frequency, types and amount smoked) and the amount already smoked on the day of survey and in the past hour before survey. A range of physical measurements were undertaken by trained staff, including height, weight, bio-impedance, blood pressure, heart rate, lung function and exhaled carbon monoxide. Blood samples were collected from each participant. All the participants are now being followed up for cause-specific mortality as well as any episodes of hospitalization, using routine mortality and health insurance data.

Central ethical approval was obtained from the University of Oxford, the China National Center for Disease Control and Prevention (CDC) and the institutional research boards at the local CDCs in the 10 regions prior to start of the survey. All participants gave their written informed consent.

### Measurement of COex

COex level was measured by a hand-held battery operated meter (MicroCO meter, CareFusion, UK). The device also estimates blood carboxyhaemoglobin (COHb, %) level based on its high correlation with COex level.^19^ The meter was calibrated regularly using a calibration gas of up to 20 ppm CO in air. Based on manufactorer’s recommendation, participants were asked to inhale fully and hold their breath for 20 s before each measurement, and then blow slowly and fully into the mouthpiece adapter of the meter. In rare circumstances (e.g. physical frailty due to old age or presence of chronic respiratory conditions), participants were allowed to hold breath for a shorter time period but no less than 10. Spirometry testing of FEV_1_ and FVC was conducted using a hand-held spirometer (Micro Medical Ltd, UK).

The present study excluded the participants with extreme values of COex level (exceed the range of 1–99 ppm) and those whose COex levels were measured earlier than 07:00 am and later than 10:59 pm (*n* = 106, 0.02% of whole study population), since these values may well reflect on-site data entry error. The cut-point of 7 ppm for COex was chosen to define abnormal levels of exposure to smoking and indoor household sources of CO. This was partly based on the sensitivity and specificity analyses of the collected COex data among current smokers who smoked on the day of collection and never smokers (sensitivity = 82.6%, specificity = 82.8%), although the study was not designed to define the COex cutoff value for smokers and never smokers.

### Statistical analysis

Analyses for the association between COex level and smoking habit were conducted separately for men and women because of the large sex differences in smoking prevalence. To avoid any potential confounding by smoking, all analyses on household air pollution and respiration-related health outcomes were restricted to never smokers. Exposure to household air pollution was evaluated according to five main sources: types of fuel used for cooking on the day of survey, installation of chimney/extractor in kitchen, types of coal used when stove burning indoor all day long, types of fuel used for heating in winter and whether the dwelling tending to be ‘coal-smoky’ in winter. Because of the short half-life of CO in the blood and likely seasonal variation in living conditions, specific subgroup analyses were performed according to both the time of the day and the season when the survey was carried out.

As the distribution of COex was positively skewed, COex values were logarithm-transformed. Adjusted geometric mean values and 95% confidence intervals (CI) of COex concentrations were calculated using the linear least squares regression method. A logistic regression model was used to estimate the odds ratio (OR) of various respiratory conditions according to different levels of COex among never smokers, adjusting simultaneously for age at survey, sex, education and other potential confounding factors. All analyses were performed using SAS 9.2 software.

## Results

The overall arithmetic mean (SD) COex level was 7.8 ppm (2.6) for men and 3.3 ppm (2.3) for women, and the overall geometric means were 7.2 and 3.1 ppm, respectively. Geometric mean COex level was somewhat higher in rural than in urban areas and decreased gradually with increasing age among men, but varied only slightly with age among women (Table 1). Men with higher household income and, especially, higher education had lower COex levels, but no such associations were seen in women. In both sexes, the COex level increased steadily with time of survey until about 6 pm (Supplementary Figure S1, available as Supplementary data at *IJE* online), and it also varied slightly with the season, especially among men.

**Table 1:** Geometric mean exhaled carbon monoxide (COex) level by certain baseline characteristics among 512 785 participants

Baseline characteristics	Men		Women	
	No.	Mean (95% CI) COex, ppm	No.	Mean (95% CI) COex, ppm
Age group in years^a^				
30–39	29 590	8.4 (8.3–8.5)	48 201	3.2 (3.2–3.2)
40–49	59 221	8.8 (8.8–8.9)	93 503	3.2 (3.2–3.2)
50–59	63 700	7.8 (7.8–7.9)	93 815	3.3 (3.2–3.3)
60–69	41 317	6.2 (6.2–6.3)	50 436	3.2 (3.2–3.2)
70–79	16 348	5.1 (5.1–5.2)	16 654	3.1 (3.1–3.1)
Region^b^				
Urban	91 338	6.7 (6.3–6.7)	134 839	3.0 (3.0–3.0)
Rural	118 838	7.6 (7.6–7.7)	167 770	3.4 (3.4–3.4)
Educational level^c^				
No formal school	18 654	7.5 (7.4–7.7)	76 543	3.1 (3.1–3.1)
Primary school	70 085	7.4 (7.3–7.4)	95 080	3.2 (3.2–3.2)
Middle school	68 162	7.4 (7.4–7.5)	76 728	3.3 (3.3–3.3)
High school	36 723	6.9 (6.8–6.9)	40 797	3.3 (3.2–3.3)
College/university	16 552	5.7 (5.7–5.8)	13 461	3.1 (3.1–3.1)
Household income (Yuan/year)^c^				
<10 000	54 712	7.3 (7.3–7.4)	90 061	3.2 (3.2–3.3)
10 000–19 999	59 544	7.3 (7.2–7.3)	89 443	3.2 (3.2–3.2)
20 000–34 999	53 395	7.0 (7.0–7.1)	73 310	3.1 (3.1–3.2)
≥35 000	42 525	6.9 (6.8–6.9)	49 795	3.2 (3.2–3.2)
Overall	210 176	7.2 (7.2–7.3)	302 609	3.1 (3.1–3.1)

^a^Adjusted for area.

^b^Adjusted for age at survey, and rural/urban area.

^c^Adjusted for age at survey and area.

**Figure 1:**
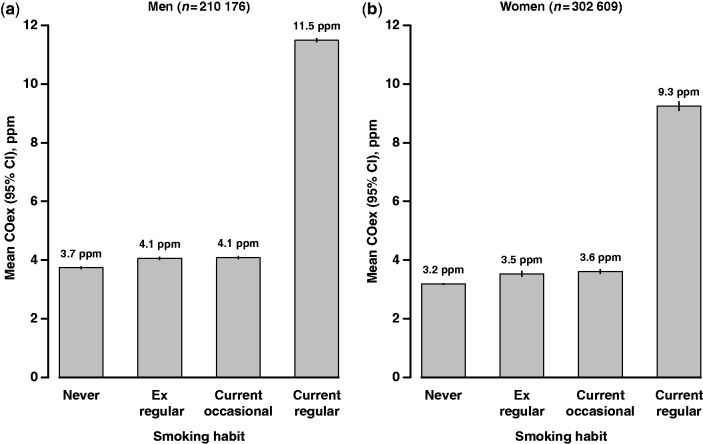
Geometric mean COex level by baseline self-reported smoking habit among 512 785 men and women. Estimates adjusted for age at survey, area, time of day and season

In both sexes, current smokers were much more likely to have increased levels of COex than never smokers (Table 2), with the unadjusted proportion having COex ≥14 ppm being about 9-fold higher in male smokers (50.2% vs 5.5%) and 5-fold higher in female smokers (31.5% vs 5.7%). After adjusting for age at survey, area, time of day and season, there was a 3-fold difference in geometric mean COex between current regular smokers and never smokers for both men (11.5 vs 3.7 ppm) and women (9.3 vs 3.2 ppm) (Figure 1).The adjusted mean COex level in ex-regular smokers and occasional smokers was similar to that in never smokers.

**Table 2:** Distribution of COex level by smoking habit in men and women

	Men, %				Women, %			
COex (ppm)	Never smoker (*n* = 30 275)	Ex regular smoker (*n* = 27 914)	Current occasional smoker (*n* = 23 625)	Current regular smoker (*n* = 128 362)	Never smoker (*n* = 287 274)	Ex regular smoker (*n* = 2645)	Current occasional smoker (*n* = 5531)	Current regular smoker (*n* = 7159)
1.0 – 7.0	79.9	78.0	70.9	20.8	83.0	85.4	80.8	35.7
7.0 – 14.0	14.5	16.0	20.4	29.1	11.3	11.6	13.3	32.8
≥ 14.0	5.5	6.0	8.7	50.2	5.7	3.1	6.0	31.5
Median(IQR)	4.0(2.0–6.0)	4.0(2.0–6.0)	4.0(3.0–7.0)	14.0(7.0–21.0)	3.0(2.0–5.0)	3.0(2.0–5.0)	3.0(2.0–6.0)	9.0(5.0–15.0)

IQR, interquartile range.

**Figure 2:**
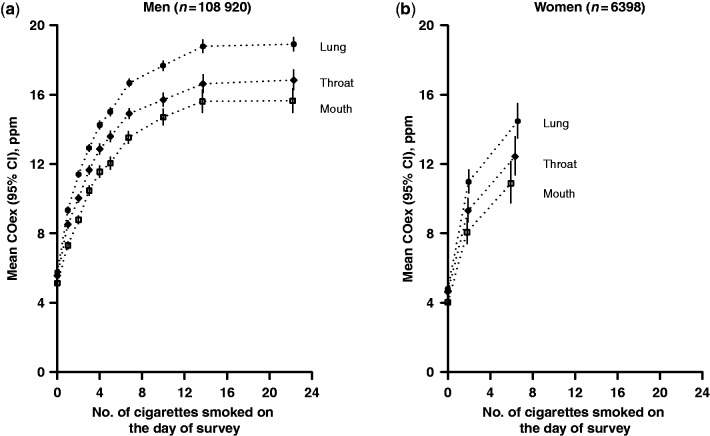
Geometric mean COex level by number of cigarettes smoked on the day of survey and level of inhalation, among male and female current regular cigarette smokers. Estimates adjusted for age at survey, area, time of day and season

Overall, smokers of manufactured cigarettes(85% of the male and 89% of the female smokers) had higher mean COex levels than smokers of other forms of tobacco (e.g. hand-rolled cigarette, pipe or water-pipe, or mixed types) (Table 3). Among those who smoked manufactured cigarettes only, mean COex levels increased substantially with the number of cigarettes smoked on the day of survey, in the previous hour, or during a usual day. However, for number of cigarettes smoked on the day of survey (at least), the mean COex did not increase any further above about 12–14 cigarettes in men (Figure 2). For women, the association also seemed to be levelling off, but very few smoked >10 cigarettes per day. In men, mean COex levels showed an inverse association with the age at which they first began smoking. In both sexes, COex level was also positively associated with the level of self-reported inhalation, for example being 11% higher overall in men who reported inhaling to the throat than in men who reported inhaling to the mouth only, and a further 11% higher in men who inhaled to the lung (Table 3). Higher mean COex level for deeper inhalation was seen with every number of cigarettes smoked per day (Figure 2).

**Table 3:** Geometric mean COex level by certain smoking characteristics among 135 521 current regular smokers

Smoking characteristics	Men		Women	
	No.	Mean COex (95% CI), ppm	No.	Mean COex (95% CI), ppm
Any tobacco smoker^a^				
Hand-rolled cigarettes only	12 615	10.8 (10.0–11.6)	631	8.7 (7.4–10.1)
Pipe or water-pipe only	1 984	13.1 (12.2–14.2)	40	12.3 (9.5–15.8)
Mixed types	4 843	14.3 (13.3–15.4)	90	11.1 (9.1–13.6)
Manufactured cigarettes only	108 920	15.1 (14.1–16.2)	6398	13.1 (11.3–15.1)
Manufactured cigarette smokers only				
No. smoked on the day of survey^b^				
0	15 575	5.5 (5.4–5.5)	1428	4.5 (4.3–4.7)
1–2	20 503	9.3 (9.2–9.4)	2504	8.9 (8.5–9.3)
3–4	23 650	12.2 (12.1–12.3)	1436	11.5 (10.9–12.0)
5–8	27 154	14.4 (14.3–14.5)	757	12.8 (12.1–13.6)
≥9	22 038	16.5 (16.3–16.6)	273	13.4 (12.3–14.7)
No. smoked in past hour^b^^,^^c^				
0	32 265	11.2 (11.1–11.2)	2570	9.1 (8.7–9.5)
1	38 240	13.1 (13.0–13.2)	2122	10.5 (10.0–11.1)
≥2	22 840	14.9 (14.8–15.0)	278	12.0 (11.0–13.1)
No. smoked in usual day^a^				
1–4	5 672	12.5 (11.6–13.4)	1498	10.7 (9.3–12.3)
5–14	30 356	14.1 (13.1–15.1)	3455	12.3 (10.7–14.1)
15–24	54 980	15.8 (14.7–16.9)	1334	12.7 (11.1–14.5)
≥25	17 912	16.1 (15.0–17.2)	111	13.7 (11.5–16.2)
Age started smoking (years)^d^				
<20	36 832	17.4 (16.3–18.7)	1684	12.7 (11.0–14.5)
20–24	41 605	16.6 (15.5–17.8)	1162	12.9 (11.3–14.8)
25–30	14 918	15.8 (14.7–16.9)	811	12.5 (10.9–14.4)
≥30	15 565	14.3 (13.4–15.4)	2741	12.5 (10.9–14.2)
Level of inhalation^e^				
To mouth only	23 949	14.1 (13.2–15.1)	1920	11.3 (9.9–13.0)
To throat only	30 508	15.7 (14.7–16.8)	1914	13.1 (11.4–14.9)
Lung	54 463	17.5 (16.4–18.8)	2564	13.7 (12.0–15.6)

^a^Adjusted for age at survey, area, time of day, season, no. of cigarettes smoked on day of survey and level of inhalation.

^b^Adjusted for age at survey, area, time of day, season and level of inhalation.

^c^Restricted to participants who had smoked 1 cigarette or more on the day of survey.

^d^Adjusted for area, time of day, season, no. of cigarettes smoked on the day of survey and level of inhalation.

^e^Adjusted for age at survey, area, time of day, season and no. of cigarettes smoked on the day of survey.

Among never smokers, the proportion having a COex level ≥7 ppm was much higher in rural (24%) than in urban areas (9%) after adjusting for age at the baseline survey, time of day, season, gender and education. In rural areas, 40% of never smokers were exposed daily to passive smoking, 54% used coal or wood as fuel for cooking, 33% had no chimney in the kitchen, 61% had a stove burning slowly indoors all day long and 11% used coal or wood as fuel for heating in winter. The corresponding figures in urban areas were all substantially lower, being 30%, 5%, 12%, 5% and 2%, respectively. In both urban and rural areas the proportion having an elevated COex level increased with greater weekly duration of exposure to passive smoking (Table 4). Similarly, the proportion of elevated COex levels was appreciably higher among those who reported having no chimney in the kitchen, using coal as fuel for cooking, having longer exposure to cooking on the day of survey, using smoky coal or brick/coalite as fuel while having a stove burning indoors all day long, or using coal or wood as fuel for heating in winter. In general, the difference in COex between exposed and unexposed groups was greater in spring and winter than in summer and autumn seasons, especially in rural areas. After controlling for age, area, time of day, season and education, these patterns were similar in men and women (Supplementary Table S1, available as Supplementary data at *IJE* online).

**Table 4:** Proportion of people with elevated COex level by exposure to various sources of indoor air pollution among 317 549 never smokers in urban and rural areas

Types of exposure	Urban				Rural			
	No.	COex ≥7 ppm		*P*-value	No.	COex ≥7 ppm		*P*-value
		No.	(%^a^)			No.	(%^a^)	
Current daily exposure to passive smoking								
No	25 269	2339	(9.4)		38 555	7551	(22.2)	
Yes	43 624	4938	(11.2)	<0.0001	69 079	16 575	(22.5)	0.15
Hours of exposure per week								
1–10	21 470	2218	(10.1)		38 310	6322	(21.2)	
11–20	8625	989	(10.9)		10 324	2306	(23.6)	
≥21	13 529	1731	(13.5)	<0.0001	20 445	7947	(29.3)	<0.0001
Having chimney in kitchen								
Yes/no cooking facility	128 859	15 194	(11.0)		114 538	13 925	(21.4)	
No	16787	1045	(12.6)	<0.0001	57 365	24 593	(24.4)	<0.0001
Cooking on the day of survey^b^								
Cooking fuel								
Electricity/gas	45 849	5639	(11.7)		9001	559	(20.4)	
Wood	5732	157	(7.7)		34 909	2556	(17.0)	
Coal	831	199	(20.6)	<0.0001	58 609	23 462	(32.1)	<0.0001
Duration of cooking (minutes)								
1–60	51 596	5825	(11.3)		94991	22 081	(25.3)	
≥61	816	170	(17.5)	<0.0001	7528	4496	(33.4)	<0.0001
Type of coal used when indoor stove burning all day long^b^^,^^c^								
Smokeless coal	1699	99	(6.7)		47 667	6536	(24.6)	
Smoky coal	1611	217	(8.6)		7189	931	(30.4)	
Coal brick/coalite	3287	247	(9.5)	0.005	50 271	24 997	(36.9)	<0.0001
Season when surveyed								
Spring	44 185	5582	(12.9)		52 601	12 969	(23.3)	
Summer	36 779	4055	(10.8)		41 634	6130	(11.4)	
Autumn	37 565	3763	(9.5)		43 843	7324	(17.9)	
Winter	27 117	2839	(11.1)	<0.0001	33 825	12 095	(40.4)	<0.0001
Type of fuel used for winter heating^b^^,^^d^								
Electricity/gas/central heating	10 148	1695	(12.9)		1796	405	(45.6)	
Wood	436	87	(19.7)		6658	2214	(47.7)	
Coal	2419	342	(30.0)	<0.0001	12 704	7923	(51.6)	0.09
Current house ‘coal-smoky’ (among those surveyed in winter)									
No	24 831	2551	(10.6)		11 294	976	(32.7)	
Yes	2286	288	(9.1)	0.15	22 531	11 119	(37.3)	0.0007

*P*-values are for heterogeneity or trend test, as appropriate.

^a^Adjusted for age at survey, sex, area, time of day, season, educational level, fuel used for heating in winter, chimney availability, cooking on the survey day, stove used inside the house and ‘coal-smoky’ in winter.

^b^Restricted to participants who reported specified type of fuel used for cooking, stove or heating.

^c^Restricted to participants who kept a stove burning slowly all day long inside house.

^d^Restricted to participants who were surveyed in winter and heated their houses in winter.

**Figure 3:**
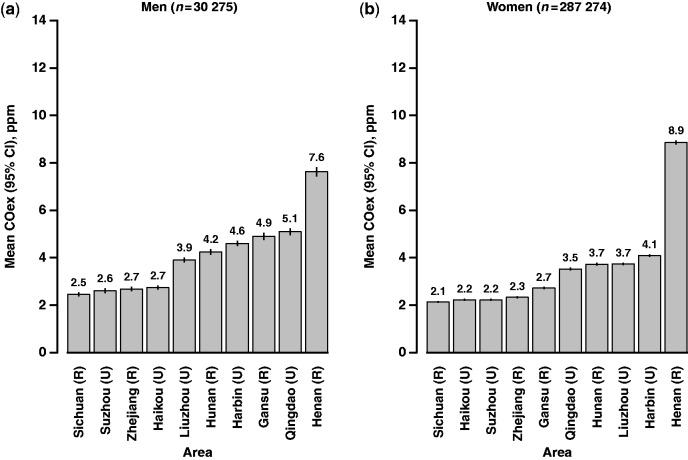
Regional variations of geometric mean COex level among male and female never smokers. Estimates adjusted for age at survey, time of day, season and educational level. Letter R denotes rural area and U denotes urban area

Across the 10 study areas, there was about a 3-fold variation in mean COex level among male never smokers, and about a 4-fold variation among female never smokers, with, for both sexes, the highest mean COex levels seen in Henan (central China) and the lowest in Sichuan (southwest China) (Figure 3). The regional variation in mean COex was much larger during winter, with ranges of 1.8–13.9 ppm in men and 1.6–15.8 ppm in women, compared with 3.1–4.9 ppm and 2.1–5.3 ppm, respectively, in summer (Supplementary Figure S2, available as Supplementary data at *IJE* online).

Among never smokers, about 2% had reported having chronic respiratory symptoms and the proportions having a prior history of doctor-diagnosed tuberculosis or of emphysema/chronic bronchitis were 1.2% and 2.2%, respectively. Based on the measured lung function, 4.1% could be classified as having airflow obstruction (i.e. FEV_1_/FVC <0.7) at baseline. There was a strong positive association between COex level and the prevalence of various chronic respiratory symptoms or conditions (Table 5). For airflow obstruction, the adjusted ORs were 1.38 (95% CI 1.31–1.45) and 1.65 (1.52–1.80), respectively, for those with measured COex of 7–14 and ≥14 ppm (*P*_trend_ <0.001). The positive association between COex and risk of chronic respiratory symptoms or conditions was observed in both urban and rural areas (Table 5) but generally appeared to be less pronounced in men than in women (Supplementary Table S2, available as Supplementary data at *IJE* online).

**Table 5:** Prevalence of self-reported or measured chronic respiratory conditions and odds ratios, by COex level, among 317549 never smokers

COex Category (ppm)	No. of never smokers	Cough frequently for ≥3 months		Cough up sputum in morning for ≥ 3 months		Self-reported tuberculosis		Self-reported emphysema/ Chronic bronchitis		Air flow obstruction (ie, FEV1/FVC < 0.7) at baseline	
		No. (%^a^)	OR^a^ (95% CI)	No. (%^a^)	OR^a^ (95% CI)	No. (%^a^)	OR^a^ (95% CI)	No. (%^a^)	OR^a^ (95% CI)	No. (%^a^)	OR^a^ (95% CI)
All areas											
1.0 – 7.0	262 792	6 055 (2.2)	1.00 (0.95–1.05)	5 453 (2.0)	1.00 (0.95–1.05)	2 999 (1.2)	1.00 (0.95–1.06)	5 982 (2.1)	1.00 (0.96–1.05)	10 946 (3.8)	1.00 (0.96–1.04)
7.0 – 14.0	36 859	909 (2.8)	1.25 (1.18–1.33)	859 (2.6)	1.29 (1.21–1.37)	652 (1.4)	1.21 (1.13–1.30)	766 (2.5)	1.16 (1.09–1.25)	1 390 (5.2)	1.38 (1.31–1.45)
≥14.0	17898	429 (2.9)	1.30 (1.17–1.44)	438 (2.9)	1.47 (1.32–1.63)	261 (1.5)	1.28 (1.12–1.46)	282 (2.6)	1.20 (1.06–1.36)	700 (6.2)	1.65 (1.52–1.80)
*P for trend*		*0.01*		*<0.0001*		*<0.0001*		*<0.0001*		*0.0009*	
Urban											
1.0 – 7.0	129407	2352 (1.8)	1.00 (0.95–1.05)	2286 (1.8)	1.00 (0.95–1.06)	1923(1.6)	1.00(0.95–1.06)	3197 (2.5)	1.00 (0.95–1.05)	4460 (3.3)	1.00 (0.96–1.04)
7.0 – 14.0	13431	298 (2.3)	1.27 (1.13–1.42)	312 (2.3)	1.29 (1.16–1.45)	371(1.9)	1.18(1.07–1.31)	331 (2.5)	1.03 (0.92–1.15)	368 (3.9)	1.19 (1.07–1.32)
≥14.0	2808	68 (2.4)	1.35 (1.06–1.71)	69 (2.4)	1.35 (1.06–1.71)	84(2.1)	1.34(1.07–1.67)	72 (2.8)	1.13 (0.89–1.43)	73 (3.8)	1.15 (0.91–1.46)
*P for trend*		*<0.0001*		*<0.0001*		*0.0004*		*0.33*		*0.005*	
Rural											
1.0 – 7.0	133385	3703 (2.6)	1.00 (0.94–1.06)	3167 (2.2)	1.00 (0.94–1.07)	1 076(0.8)	1.00(0.91–1.10)	2785 (1.9)	1.00 (0.93–1.07)	6486 (4.2)	1.00 (0.95–1.05)
7.0 – 14.0	23428	611 (3.2)	1.24 (1.16–1.34)	547 (2.8)	1.29 (1.20–1.40)	281(1.0)	1.25(1.12–1.39)	435 (2.4)	1.31 (1.20–1.43)	1022 (6.2)	1.52 (1.44–1.61)
≥14.0	15090	361 (3.2)	1.27 (1.13–1.43)	369 (3.3)	1.51 (1.34–1.70)	177(1.1)	1.29(1.09–1.52)	210 (2.3)	1.26 (1.08–1.46)	627 (7.5)	1.88 (1.71–2.06)
*P for trend*		*<0.0001*		*0.0004*		*0.002*		*<0.0001*		*<0.0001*	

^a^adjusted for age at survey, sex, area, season, time of day and educational level.

## Discussion

This is the first large-scale study in China of the lifestyle and environmental determinants of COex levels. It involved 10 geographically diverse urban and rural regions and shows that smoking is the main cause of elevated COex level in adult Chinese, with the geometric mean COex level being about 3-fold higher in male and female current smokers compared with never smokers. There was a clear dose-response relationship between measured COex level and the amount smoked up to about 12–14 cigarettes per day, and with depth of inhalation. Among never smokers, mean COex levels varied greatly across the 10 study regions, especially during winter, and exposure to passive smoking and various sources of household air pollution was associated with increased COex levels. Moreover, COex level was associated with the prevalence of airflow obstruction and various other chronic respiratory symptoms and conditions.

In the past few decades several large nationwide surveys have consistently shown that smoking remains a largely male phenomenon in China, with about two-thirds of adult men being current smokers compared with less than 5% in women.^20^^,^^21^ A recent report in China showed that 53% of men and 2.4% of women aged 15 years and older were current tobacco smokers.^13^ However, none of these previous surveys used objective measures to validate self-reported smoking history, although since smoking is still regarded as a socially acceptable habit for adults in China (particularly in men), self-report may be reasonably reliable. Using COex as a short-term objective measure of smoking, the present study provides further evidence about the widespread use of tobacco in Chinese men and the comparatively low rate of smoking in Chinese women. Overall, 74% of men in the study had previously been regular smokers, a figure similar to that reported in a nationally representative survey of 220 000 adult men during the early 1990s.^22^ Although our study is not intended to be nationally representative, it is notable that 13% of men were ex-smokers, as assessed by COex level, as opposed to only 1% in the previous study.^22^ With good characterization of smoking habits, as well as of the amount smoked and the degree of inhalation, the present study is well placed to assess the hazards of smoking and the benefits of stopping in Chinese adults over the next few decades.

A number of studies from high-income countries have reported on the association of COex with exposure to passive smoking, but the results have been inconsistent, with some showing no clear association,^23^ whereas others, in particularly those involving occupational cohorts (e.g. bar waiters), reporting strong positive associations.^24–26^ Unlike active smoking, reliable assessment of exposure to passive smoking using COex will depend not only on the intensity of exposure (e.g. duration and amount of smoke) but also the environment in which exposure occurred (e.g. room size, ventilation).^27^ During the past few decades, the smoking rate has been declining rapidly in most Western populations, and legislation has also been introduced in many countries to restrict or ban smoking in public places. Consequently fewer and fewer non-smokers are exposed to passive smoking at home or in the workplace. In China, the situation is quite different, with over one-third of non-smokers in the general population aged 15 years or older being exposed to passive smoking daily.^13^^,^^28^ In the present study of adults aged 30 years or above, about 60% of never smokers reported being exposed to passive smoking on most days, with almost 40% of women exposed daily. Despite the relatively short half-life of CO, the proportion having elevated COex levels increased with increasing duration of exposure to passive smoking. This suggests that, in places such as China where smoking is still prevalent, COex could be used (in conjunction with questionnaires) as a surrogate biomarker for assessing the degree of exposure to passive smoking.

Besides tobacco smoke, incomplete combustion of biomass fuel (mainly wood and crop residues) and coal are the main indoor household sources for elevated CO.^29^ Despite recent economic development, coal and wood remain the most commonly used fuels for cooking and heating in many parts of China.^30^ In the present study, indoor household air pollution was assessed by questionnaire and focused primarily on usual patterns of exposure rather than exposure immediately before the survey, so the information collected may be incomplete or poorly correlated with the actual level of exposure at the time when the participants were surveyed. Despite this, a number of parameters related to the extent of exposure to household air pollution showed independent associations with COex, even though the effect size tended to be modest. Since the analyses were restricted to never smokers, potential confounding by smoking can be excluded. The association appeared most pronounced in rural areas, during winter, and with exposure to coal brick or coalite, thereby characterizing a principal source of indoor household air pollution in China.

The daily temporal trends and seasonal variations in COex level in the present study were consistent with findings reported in previous studies.^23^^,^^31^ This can be explained mainly by habitual daily cooking times and the form of house heating in the northern region.^32^ The relatively high COex level observed in four study areas (Henan, Harbin, Gansu and Qingdao) can be partly explained by their location in northern China where heating in winter and domestic use of coal/wood is very common. Despite this, much of the large regional variation in measured COex level among never smokers is still unaccounted for, particularly during winter. This may reflect, in part, the incomplete measures of household air pollution in the present study and, in part, the limited value of using a single COex measure to assess long-term exposure to household air pollution. Given these limitations, no attempt has been made in the present study to develop an overall exposure index from various sources. Similarly, no analysis has been made to try to assess the extent to which the proportion of variation in COex by age, gender or region could potentially be explained by exposures to smoking and household air pollution. To assess the effects of household air pollution more accurately, direct measurement of indoor air pollution (particularly with personal exposure monitoring) might provide a better estimate. Given the likely seasonal and daily variation in exposure, such measurements would need to be made for an extended period of time and at different time-points during the year.

Despite the 3-fold difference in the mean COex level between smokers and never smokers, the mean level of measured COex in CKB participants was only about half of that seen in Western populations for both smokers (∼10 in CKB vs ∼20 ppm) and never smokers (∼4 in CKB vs ∼8 ppm).^2^ Similar findings have also been reported in other Asian populations living in both Asia and Western countries.^9^^,^^12^^,^^33^ A number of factors may contribute to the relatively lower COex levels among Asian smokers, including number of cigarettes smoked, degree of inhalation as well as background level of CO from other sources. Moreover, because CO is absorbed through lung alveoli, there may be ethnical differences in alveolar ventilation or CO diffusion capacity of lung between different populations. If this were true, it could also help explain in part the difference in the mean levels of COex between Chinese and Western populations.

Although elevated CO level has previously been shown to be associated with increased risk of myocardial infarction in smokers,^34^ few studies have investigated the health effects of low-level exposure to ambient CO on respiratory and other diseases among never smokers. Among never smokers, the present study found a positive association between mean COex and the prevalence of various respiratory conditions, including airflow obstruction based on measured lung function (*P* <0.01). Whereas the association could be largely causal, it is also possible that individuals with impaired lung function may not be able to clear CO from their lungs as effectively. If these findings were confirmed in similar prospective analyses of disease incidence, it would help to establish the value of COex, despite its short half-life, as a simple and practical biomarker for assessing exposure to indoor air pollution and its relation to long-term health effects.

In summary, the present study has provided the first large-scale evidence from China that the level of COex is, on average, much higher in smokers than in never smokers and that it also correlates strongly with the amount of tobacco smoked and the degree of inhalation. Routine measurement of COex should be considered in future epidemiological studies of smoking as well as in smoking cessation programmes in China. Given the widespread use of biomass and coal for cooking and heating in many parts of rural China and the potential adverse health consequences, it may be appropriate to use COex as a simple surrogate biomarker to assess the overall burden of the exposure from such sources and its contribution to related health consequences. Continuing follow-up of the present study participants will soon provide large-scale prospective evidence on the relevance of such measures to subsequent health outcomes.

## Supplementary Data

Supplementary data is available at *IJE* online.

## Funding

The baseline survey and first re-survey in China were supported by a research grant from the Kadoorie Charitable Foundation in Hong Kong; follow-up of the project during 2009–14 is supported by the Wellcome Trust in the UK (grant 088158/Z/09/Z), and the National Key Technology Research and Development Program in the 12th Five-Year Plan, Ministry of Science and Technology, China; the Clinical Trial Service Unit and Epidemiological Studies Unit (CTSU) at Oxford University also receive core funding for the project from the UK Medical Research Council, the British Heart Foundation and Cancer Research UK.

## Supplementary Material

Supplementary Data
