# Supplementary material for: Exhaled carbon monoxide and its associations with smoking, indoor household air pollution and chronic respiratory diseases among 512 000 Chinese adults
Source: Int J Epidemiol. 2013 Sep 20;42(5):1464–75. doi: 10.1093/ije/dyt158 (PMC3807615; doi:10.1093/ije/dyt158)
Supplement: Supplementary Data [file supp_42_5_1464__index.html]

Exhaled carbon monoxide and its associations with smoking, indoor household air pollution and chronic respiratory diseases among 512 000 Chinese adults — Supplementary Data 

# Exhaled carbon monoxide and its associations with smoking, indoor household air pollution and chronic respiratory diseases among 512 000 Chinese adults

## Supplementary Data

files

**Files in this Data Supplement:**

- Supplementary Data - pdf file
