# Supplementary material for: Exhaled carbon monoxide and its associations with smoking, indoor household air pollution and chronic respiratory diseases among 512 000 Chinese adults
Source: Int J Epidemiol. 2013 Sep 20;42(5):1464–75. doi: 10.1093/ije/dyt158 (PMC3807615; doi:10.1093/ije/dyt158)
Supplement: Supplementary Data [file supp_dyt158_ije-2013-02-0222-File004.pdf]

SUPPLEMENTARY MATERIAL

Exhaled carbon monoxide and its associations with smoking, indoor household air pollution and chronic respiratory diseases among 512 000 Chinese adults

Qiuli Zhang[1], Liming Li [2, 3, 4], Margaret Smith[1], Yu Guo [3, 4], Gary Whitlock [1], Zheng Bian [3, 4], Om Kurmi [1], Rory Collins [1], Junshi Chen [3], Silu Lv [5], Zhigang Pang [6], Chunxing Chen [7], Naying Chen [8], Youping Xiong [9], Richard Peto [1], Zhengming Chen[1] on behalf of the China Kadoorie Biobank study collaboration

[1] Clinical Trial Service Unit and Epidemiological Studies Unit (CTSU), University of Oxford

[2] School of Public Health, Peking University, Haidan District, Beijing 100191, China

[3] Chinese Center for Disease Control and Prevention, Chang Ping District, Beijing 102206, China

[4] Chinese Academy of Medical Sciences, Dong Cheng District, Beijing 100730, China

[5] Licang Center for Disease Control, Qingdao, Shandong 266100, China

[6] Heilongjiang Center for Disease Control, Harbin, Heilongjiang 150030, China

[7] Meilan Center for Disease Control, Haikou, Hainan 570203, China

[8] Guangxi Center for Disease Control, Nanning, Guangxi 530028, China

[9] Liuyang Center for Disease Control, Liuyang, Hunan 410300, China

Table S1. Proportion of participants with elevated COex level by exposure to various sources of indoor household air pollution among male and female never smokers

Type of exposure	Men			Women		
	No.	COex ≥ 7 ppm No. (%[a])	P-value	No.	COex ≥ 7 ppm No. (%[a])	P-value
Current daily exposure to passive smoking						
No	2 393	491 (20.8)		61 431	9 399 (16.9)	
Yes	2 465	546 (21.8)	0.43	110 238	20 967 (18.1)	<0.0001
Hours of exposure per week						
1-10	1 402	276 (20.2)		58 378	8 264 (17.0)	
11-20	425	95 (22.9)		18 524	3 200 (18.5)	
≥ 21	638	175 (25.6)	0.02	33 336	9 503 (22.9)	<0.0001
Having chimney in kitchen						
Yes/no cooking facility	23 882	4 107 (19.6)		219 515	25 012 (16.5)	
No	6 393	1 963 (21.7)	0.01	67 759	23 675 (18.6)	<0.0001
Cooking on the day of survey[b]						
Cooking fuel						
Electricity/gas	2 784	424 (16.7)		52 066	5 774 (18.5)	
Wood	847	73 (13.4)		39 794	2 640 (13.9)	
Coal	1 192	509 (35.7)	<0.0001	58 248	23 152 (28.1)	<0.0001
Duration of cooking (minutes)						
1-60	4 687	936 (20.6)		141 900	26 970 (20.7)	
≥ 61	136	70 (29.9)	0.02	8 208	4 596 (27.5)	<0.0001
Types of coal used when indoor stove burning all day long[b,c]						
Smokeless coal	4 022	660 (24.4)		45 344	5 975 (23.5)	
Smoky coal	735	202 (35.0)		8 065	946 (28.3)	
Coal brick/coalite	4 205	1 955 (37.5)	<0.0001	49 353	23 289 (35.0)	<0.0001
Types of fuel used for winter heating[b, d, e]						
Electricity/gas/central heating	1 768	373 (30.2)		10 153	1 727 (33.6)	
Coal/wood	1 949	962 (41.1)	0.003	20 262	9 604 (39.1)	0.0001
Current house “coal-smokey” for those surveyed in winter						
No	4 072	574 (24.0)		32 053	2 953 (22.8)	
Yes	2 057	988 (28.5)	0.10	22 760	10 419 (26.6)	0.0002

Note: P-value for heterogeneity or trend test, as appropriate

[a] adjusted for age at survey, area, time of day, season, fuel used for heating in winter, chimney availability, cooking at the survey day, stove using inside the house and “coal-smokey” in winter

[b] restricted to those who reported specified type of fuel used for cooking, stove or heating

[c] restricted to the participants who kept a stove burning slowly all day long inside house

[d] two study areas (Haikou and Zhejiang) were not included in the analysis because of very limit number of cases (total 4 men and 24 women from these 2 areas were surveyed in winter)

[e] restricted to participants who were surveyed in winter and heated their houses in winter

Table S2. Prevalence of self-reported or measured chronic respiratory conditions and adjusted odds ratios by levels of COex among male and female never smokers

COex category	No. of never smokers	Cough frequently ≥ 3 months		Cough up sputum in morning ≥ 3 months		Self-reported tuberculosis		Self-reported emphysema/ bronchitis		Air flow obstruction	
		No.(% [a])	OR[a] (95% CI)	No.(% [a])	OR[a] (95% CI)	No.(% [a])	OR[a] (95% CI)	No.(% [a])	OR[a] (95% CI)	No.(% [a])	OR[a] (95% CI)
**Men**											
1.0-<7.0	24 205	786 (3.2)	1.00 (0.90-1.11)	798 (3.2)	1.00 (0.90-1.11)	626 (2.6)	1.00 (0.90-1.12)	795 (3.2)	1.00 (0.90-1.11)	1 213 (4.9)	1.00 (0.91-1.10)
7.0-<14.0	4 402	154 (3.8)	1.19 (1.02-1.38)	153 (3.6)	1.14 (0.97-1.33)	114 (2.5)	0.98 (0.82-1.18)	133 (3.4)	1.07 (0.91-1.27)	226 (5.9)	1.23 (1.08-1.40)
≥14.0	1 668	54 (3.5)	1.11 (0.84-1.48)	64 (4.5)	1.40 (1.08-1.82)	40 (2.9)	1.13 (0.81-1.57)	34 (3.0)	0.94 (0.66-1.33)	104 (6.5)	1.36 (1.09-1.69)
P for trend		*0.90*		*0.23*		*0.70*		*0.002*		*0.49*	
**Women**											
1.0-<7.0	238 587	5 269 (2.1)	1.00 (0.95-1.05)	4 655 (1.9)	1.00 (0.95-1.06)	2 373 (1.0)	1.00 (0.94-1.06)	5 187 (2.0)	1.00 (0.95-1.05)	9 733 (3.7)	1.00 (0.96-1.04)
7.0-<14.0	32 457	755 (2.7)	1.28 (1.20-1.37)	706 (2.5)	1.33 (1.25-1.43)	538 (1.3)	1.28 (1.18-1.38)	633 (2.4)	1.18 (1.10-1.27)	1 164 (5.2)	1.44 (1.36-1.52)
≥14.0	16 230	375 (2.8)	1.33 (1.19-1.50)	374 (2.8)	1.48 (1.32-1.66)	221 (1.4)	1.32 (1.14-1.53)	248 (2.5)	1.24 (1.08-1.42)	596 (6.3)	1.76 (1.61-1.93)
P for trend		*0.008*		*<0.0001*		*<0.0001*		*<0.0001*		*0.0002*	

[a] adjusted for age at survey, area, time of day, season and educational level

Figure S1. Geometric COex level by time of day and month among 210 176 men and 302 609 women

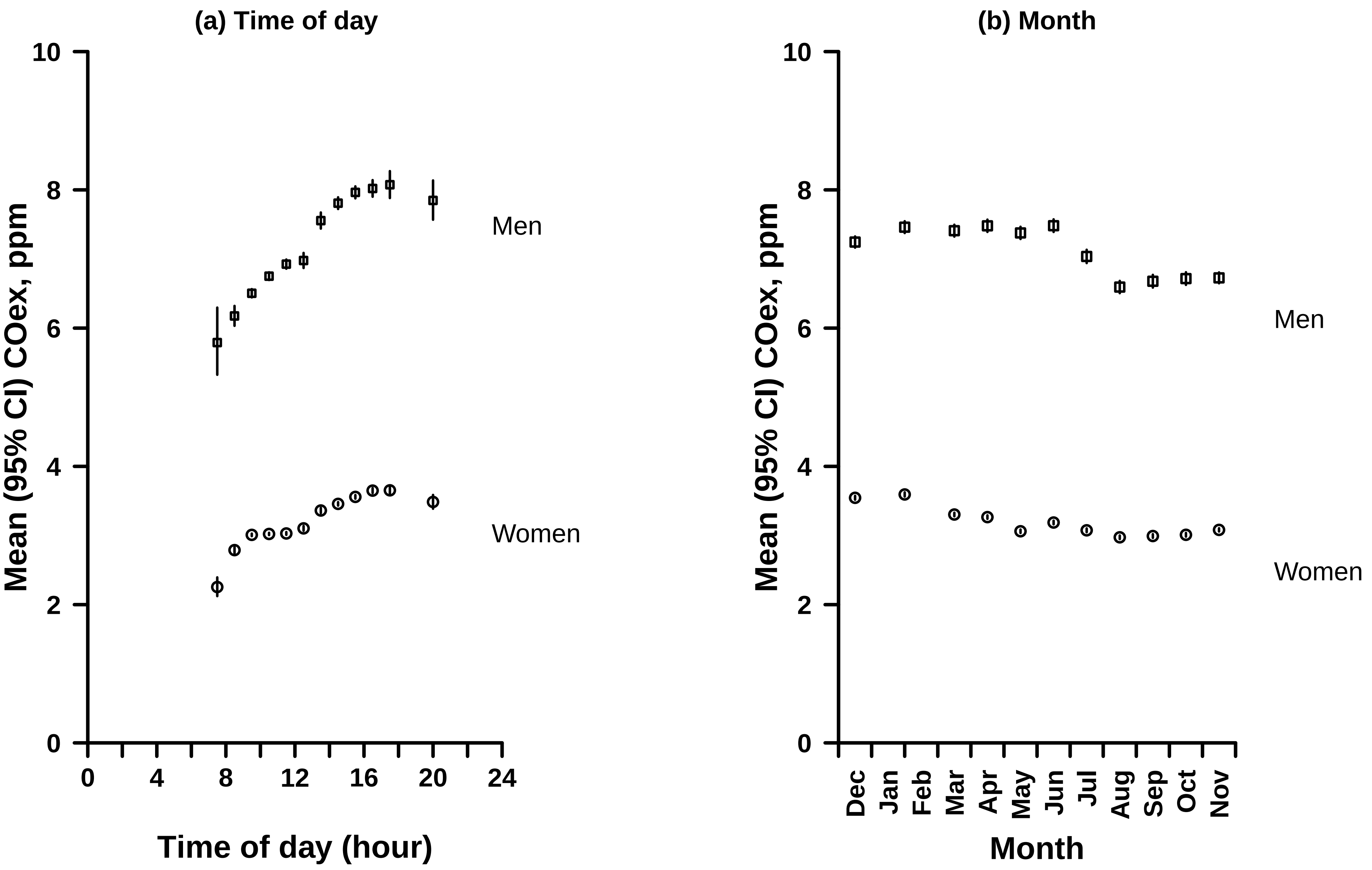

Estimates adjusted for age at survey and area

Figure S2. Regional variations of geometric mean COex level by season among male and female never smokers

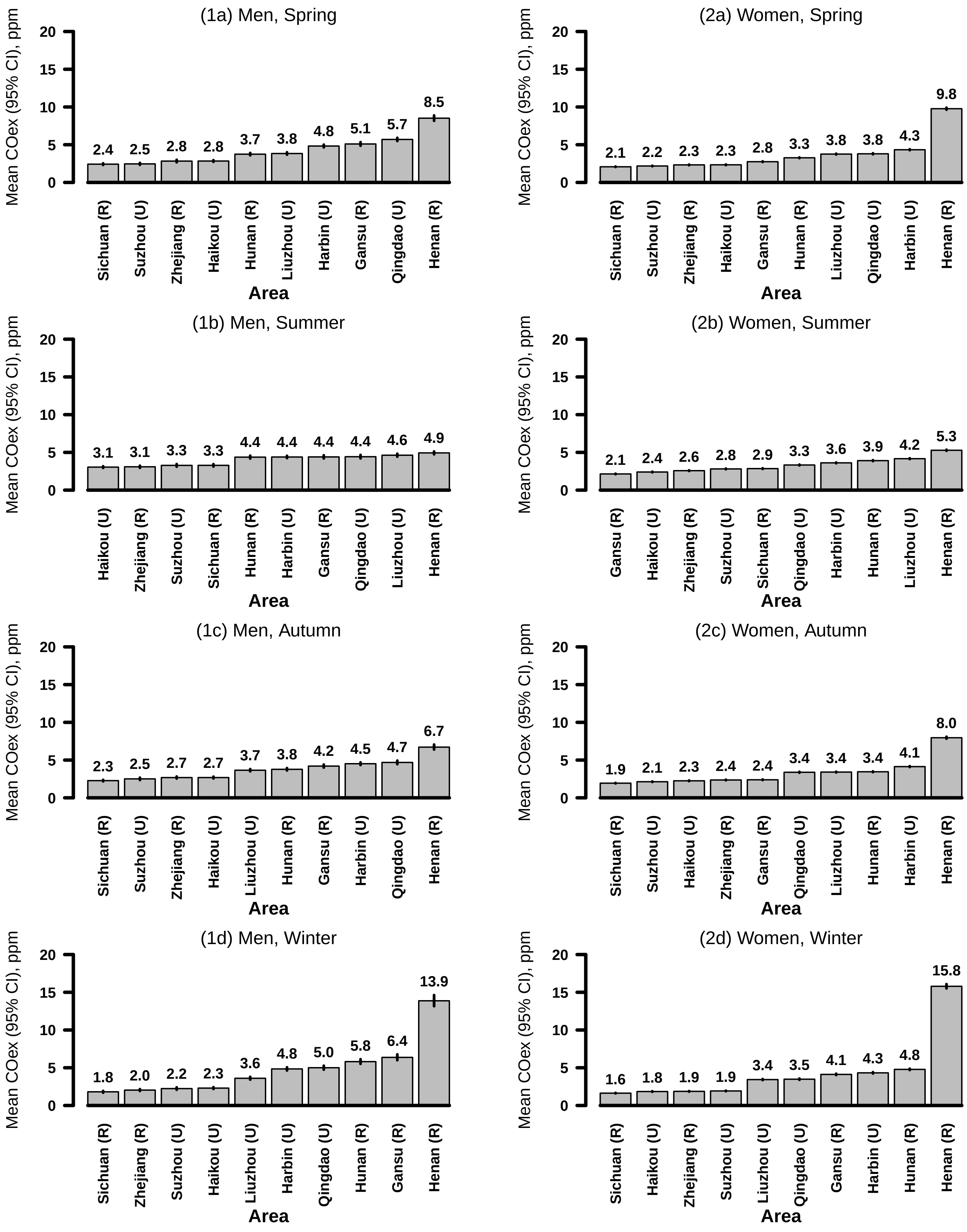

Estimates adjusted for age at survey, time of day and educational level
Abbreviation: U, urban area; R, rural area
